# Knee joint biomechanics in transtibial amputees in gait, cycling, and elliptical training

**DOI:** 10.1371/journal.pone.0226060

**Published:** 2019-12-12

**Authors:** Greg Orekhov, A. Matt Robinson, Scott J. Hazelwood, Stephen M. Klisch

**Affiliations:** 1 Mechanical Engineering Department, California Polytechnic State University, San Luis Obispo, CA, United States of America; 2 Hanger Clinic, San Luis Obispo, CA, United States of America; 3 Biomedical Engineering Department, California Polytechnic State University, San Luis Obispo, CA, United States of America; Holland Bloorview Kids Rehabilitation Hospital, CANADA

## Abstract

Transtibial amputees may experience decreased quality of life due to increased risk of knee joint osteoarthritis (OA). No prior studies have compared knee joint biomechanics for the same group of transtibial amputees in gait, cycling, and elliptical training. Thus, the goal of this study was to identify preferred exercises for transtibial amputees in the context of reducing risk of knee OA. The hypotheses were: 1) knee biomechanics would differ due to participant status (amputee, control), exercise, and leg type (intact, residual) and 2) gait kinematic parameters would differ due to participant status and leg type. Ten unilateral transtibial amputee and ten control participants performed exercises while kinematic and kinetic data were collected. Two-factor repeated measures analysis of variance with post-hoc Tukey tests and non-parametric equivalents were performed to determine significance. Maximum knee compressive force, extension torque, and abduction torque were lowest in cycling and highest in gait regardless of participant type. Amputee maximum knee extension torque was higher in the intact vs. residual knee in gait. Amputee maximum knee flexion angle was higher in the residual vs. intact knee in gait and elliptical. Gait midstance knee flexion angle timing was asymmetrical for amputees and knee angle was lower in the amputee residual vs. control non-dominant knees. The results suggest that cycling, and likely other non-weight bearing exercises, may be preferred exercises for amputees due to significant reductions in biomechanical asymmetries and joint loads.

## Introduction

Transtibial amputees may experience decreased quality of life due to increased risk of knee joint osteoarthritis (OA) [1], a degenerative disease of bone and cartilage tissues that often leads to debilitating joint stiffness and pain. Abnormal biomechanics during daily activities, including gait, is well accepted to be a risk factor for knee joint OA [2–5], and is likely related to the high prevalence of joint pain and OA among military and civilian unilateral transtibial and transfemoral amputees [1,6–10].

Transtibial amputees are more likely to develop OA in the intact knee joint than the residual (i.e. amputated) joint [1,11,12], which may be caused by preferring the intact leg as evidenced by abnormal gait biomechanics including asymmetric ground reaction forces, muscle activation patterns, and knee joint kinetics (i.e. forces and torques) between the intact and residual limbs [13–21]. Studies have shown that transtibial amputees, as compared to non-amputee controls, have increased intact knee joint forces and torques [22] and increased asymmetry in internal knee abduction torque [23,24]. Knee OA is more commonly seen in the medial compartment and, generally, high internal abduction torque increases medial compartment loading [25]. Although there is contention about the relevance of knee extension torque in OA risk [26], an *in vivo* study showed that extension torque is a significant contributor to medial contact force [27].

While prosthetic limb design has advanced in recent years, with energy storage and release (ESAR) prostheses representing the state-of-the-art in passive-elastic devices, such prostheses do not completely restore natural biomechanics of the lower limb during gait [28–30]. Studies comparing powered ankle prostheses to passive devices have shown that increasing net positive prosthetic ankle work can decrease metabolic cost of transport, increase walking speed, and potentially reduce some gait abnormalities [31–35], but the number of studies of powered prostheses is relatively low and these studies are limited to gait only. This study focuses on ESAR prostheses due to the prevalence of passive-elastic devices amongst the amputee population.

Exercise is recommended for amputees for rehabilitation and lifelong fitness sustainment [36] but there are only a few biomechanical studies for transtibial amputees in exercises other than gait. Although guidelines have been proposed for prosthetic use among transtibial amputees during cycling [37], only a few studies have addressed intact knee joint loading during cycling for this population [8,38]. In a prior cycling biomechanics study, significantly higher pedal force and work asymmetries existed between intact and residual limbs for transtibial amputees [38] and such asymmetries depended on cycling intensity and prosthetic foot stiffness [8]. Although elliptical training has been recommended during rehabilitation for this population [39], there do not appear to be any biomechanical studies of knee joint loading during elliptical training for transtibial amputees. Previous studies with non-amputee populations have shown that elliptical training, compared to walking, produces similar kinematic and kinetic patterns [40,41] and reduces knee load impulses [42]. Elliptical training is recommended alongside cycling and swimming as non-weightbearing exercises when other exercises are challenging in persons with joint OA [43].

The goal of this study was to conduct motion analysis experiments and compare knee biomechanics in gait, cycling, and elliptical training in order to identify appropriate exercises for transtibial amputees whom are at high risk for knee OA. The hypotheses were: 1) knee biomechanics would differ due to participant status (amputee, control), exercise, and leg type (intact, residual) and 2) gait kinematic parameters would differ due to participant status and leg type.

## Methods

All protocols were approved by Cal Poly's Institutional Review Board (IRB) and the U.S. Army Medical Research and Materiel Command (USAMRMC) Office of Research Protections (ORP) Human Research Protections Office (HRPO) (HRPO Log Number A-19263) and were designed to minimize risk to human subjects. Written consent was obtained prior to all experiments.

### Participant recruitment

Ten unilateral transtibial amputees (aged 18–45, body mass index [BMI] 22.3–29.6, 1.5–12.4 years post-operation, 7 males, 3 females) and ten control participants (aged 20–26, BMI 19.1–27.9, 8 males, 2 females) participated. Exclusion criteria included history of cardiovascular disease, respiratory disease, or any other metabolic disease/complication; substantial weight loss or gain over the previous six months; history of major psychiatric illness, drug abuse, or unsafe dieting practices; major medical conditions that prohibit physical activity; and pregnant women or women expecting or trying to be pregnant. After screening, eligible participants were invited to the motion analysis lab where informed consent was obtained, information forms were filled out, and amputee participants were fitted with an Energy Storage and Release (ESAR) prosthesis (Vari-Flex^®^, Ӧssur, Reykjavik, Iceland) by a certified prosthetist (AMR). One week of accommodation time was provided to amputee participants that exhibited unfamiliarity with the Vari-Flex^®^ as determined by our certified prosthetist (AMR) in qualitative alignment and gait pattern checks. However, most (8/10) amputee participants were tested without accommodation time as they used similar ESAR devices to the Vari-Flex^®^ and passed AMR’s alignment and gait pattern checks. Amputee participants all wore similar carbon fiber sockets and static pylons with no springs or dampers. Screening and health information forms indicated that all participants were relatively young, healthy, non-obese, free of disease, and had no restrictions on physical activity.

Tables 1 and 2 document critical information for amputee and control participants, respectively. Mass was measured with a scale and height measured with a stadiometer. The dominant leg for amputee participants was defined as the intact leg. The dominant leg for control participants was defined as the strongest or preferred leg and was self-reported. Age, gender, years since amputation, and co-morbidities for the intact leg were self-reported.

**Table 1:** Transtibial amputee participant characteristics.

Participant	Age	Mass [kg]	Height [m]	BMI	Intact Leg	Gender	Years Since Amputation	Co- Morbidities
1	32	74.8	1.82	22.6	L	M	11.9	Healed ACL tear
2	31	83.9	1.69	29.4	R	M	2.8	-
3	45	80.7	1.81	24.6	R	M	6.2	Charcot foot
4	32	80.6	1.78	25.4	R	M	12.4	Screws in ankle
5	32	72.4	1.80	22.3	R	M	8.1	-
6	34	92.2	1.77	29.6	L	F	10.6	-
7	29	58.4	1.54	24.7	L	F	4.2	-
8	37	82.9	1.82	25.0	L	M	8.2	Hip implant, rod in femur
9	18	76.8	1.73	25.5	R	F	1.5	-
10	32	75.0	1.81	22.8	R	M	6.8	-
**Average** **(SD)**	32.2 (6.7)	77.8 (8.9)	1.76 (0.09)	25.2 (2.6)	-	-	7.3 (3.7)	-

**Table 2:** Control participant characteristics.

Participant	Age	Mass [kg]	Height [m]	BMI	Dominant Leg	Gender
1	26	86.6	1.79	27.0	R	M
2	23	88.5	1.75	23.0	L	M
3	22	61.2	1.79	19.1	R	M
4	22	79.1	1.82	24.0	R	M
5	20	90.4	1.80	27.9	L	M
6	23	70.3	1.75	22.9	R	M
7	23	56.2	1.63	21.3	R	M
8	21	64.8	1.65	23.9	R	M
9	20	68.1	1.61	26.1	R	F
10	21	68.7	1.72	23.4	R	F
**Average** **(SD)**	22.1 (1.8)	73.4 (12.0)	1.73 (0.08)	24.4 (3.1)	-	-

### Experiments

Twelve near-infrared digital cameras (6 Owl, 3 Osprey, 2 Kestrel, 1 Eagle) (Motion Analysis Corp., Santa Rosa, CA, USA) were used capture the motion of reflective markers. Participants wore tight compression clothing that exposed as much skin as the participant was comfortable with. A modified Helen Hayes marker set was used with 32 reflective markers placed at the following anatomical landmarks: crown of the head, acromion processes, 7^th^ cervical spine, sternum, greater trochanters, anterior superior iliac spines, tops of the iliac crests, sacrum, halfway along the long axis of each thigh, lateral and medial knee condyles, fibular heads, tibial tuberosities, halfway along the long axis of each shank, lateral and medial malleoli, Achilles tendon insertion sites, and between the 2^nd^ and 3^rd^ metatarsals of both feet. When possible, markers were placed directly on skin but pelvic markers were placed on compression clothing. Prosthesis-side markers were aligned with intact-side markers as closely as possible when anatomical landmarks were inaccessible or absent.

**Fig 1:**
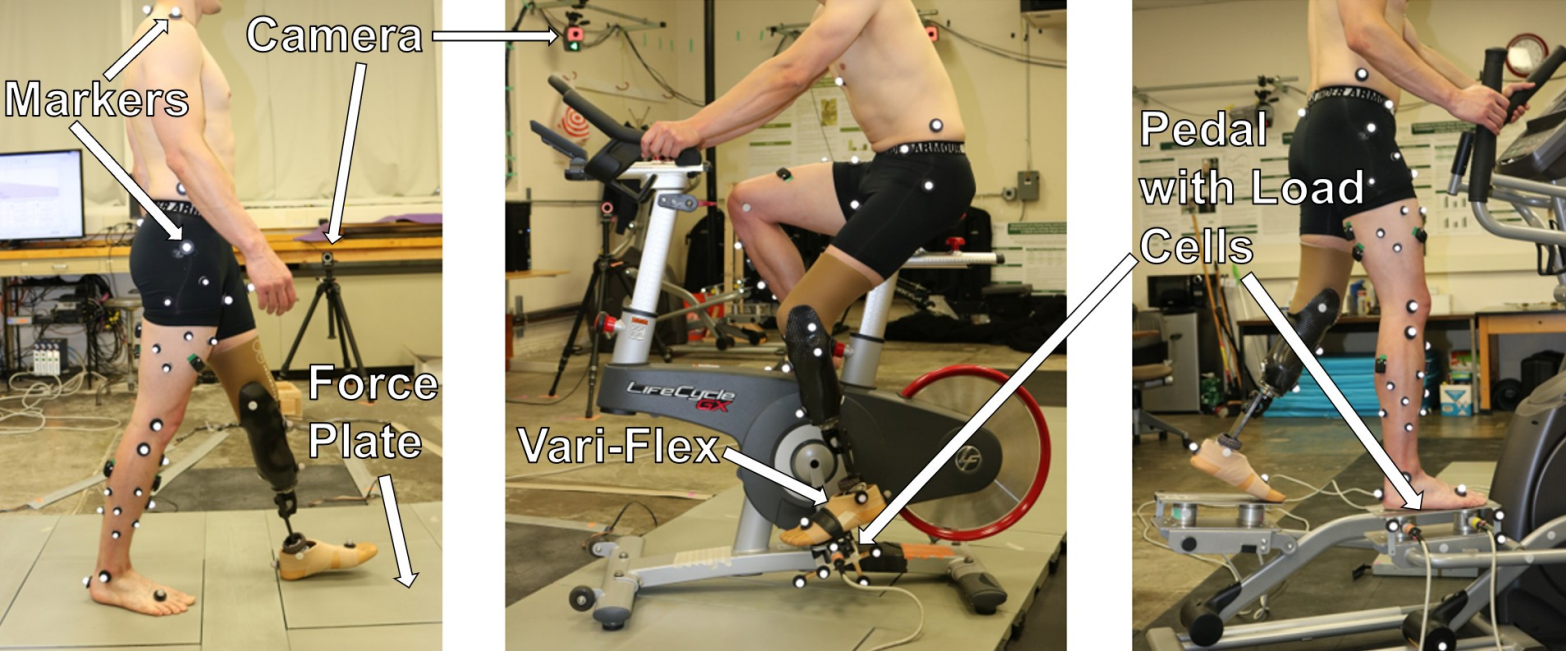
Setup for gait, cycling, and elliptical training motion analysis experiments. Digital cameras, ground force plates, reflective markers, the Vari-Flex prosthesis with foot cover, and instrumented exercise machine pedals are indicated.

Participants were asked to stand stationary in a static pose with feet at shoulder width and arms bent at the elbows. Static pose captures were used to begin the marker identification process and defined initial joint angles for later analyses. Top head and medial knee and ankle markers were removed after static captures. During gait experiments, four ground force plates (Accugait, AMTI, Watertown, MA, USA) were used to capture ground reaction forces in the anterior, lateral, and vertical directions; the free reaction torque about the vertical axis; and the center of pressure (COP) of the reaction force vector on the surface of the force platform (Fig 1). Three gait trials were collected for each participant at self-selected speeds in each direction.

During cycling and elliptical experiments, the pedals of a stationary bicycle (Lifecycle GX, Life Fitness, Schiller Park, IL, USA) and elliptical trainer (XE795, Spirit Fitness, Jonesboro, AR, USA) were instrumented with 6-axis load cells (AD2.5D, AMTI) to capture pedal reaction forces, free torque, and COP (Fig 1). Three trials for cycling and elliptical were collected at 70 revolutions per minute (RPM, range 68–72 RPM) as measured by the corresponding machine at moderate resistance settings (level 10 of 20). Once the participant reached the target speed, data for 30 seconds were collected to capture several machine cycles. Cortex software (Version 7.01, Motion Analysis) was used to interface all equipment, perform post-processing of marker data, and calculate kinematics and kinetics.

### Data processing

One full gait cycle was defined by consecutive heel strikes of the same leg (0% = 1^st^ heel strike, 100% = 2^nd^ heel strike). One cycling cycle was defined by one full crank rotation starting at top dead center (TDC; 0% = 1^st^ pass through TDC, 100% = 2^nd^ pass through TDC) [8]. One elliptical cycle was defined by one full crank rotation starting at the most anterior pedal position (APP; 0% = 1^st^ pass through APP, 100% = 2^nd^ pass through APP) [42].

The sacrum marker velocity in the direction of motion was stored and used to calculate average walking speed for each participant. The time in seconds at which cycling and elliptical pedals passed through their respective start and end of cycle were stored and used to calculate actual RPM. Cycles with RPM greater than 72 or less than 68 were eliminated from the pool of samples as kinetics vary with machine power [8]. Machine cycles were selected randomly without replacement (to avoid selecting the same cycle again) before normalizing and averaging data.

**Fig 2:**
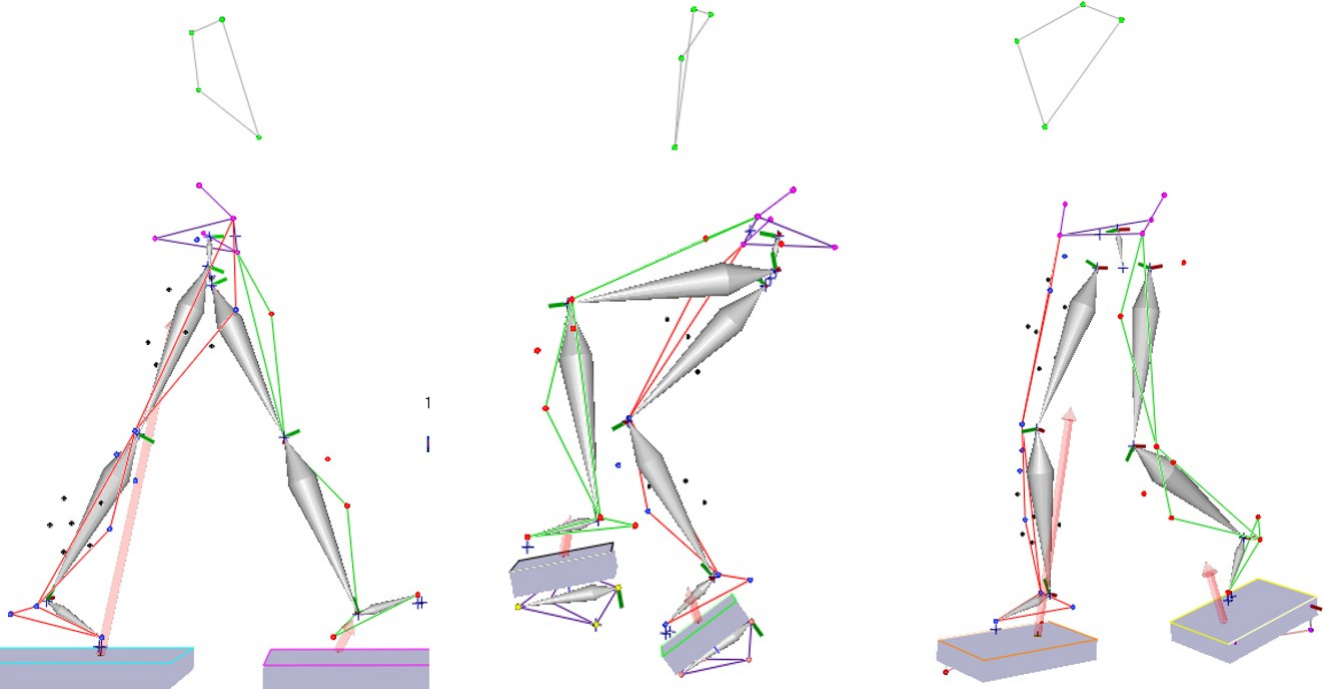
Gait, cycling, and elliptical dynamic captures in Cortex. Force plates and load cells tracking pedal mass segments are visible. Red vectors indicate magnitude and direction of reaction forces.

**Fig 3:**
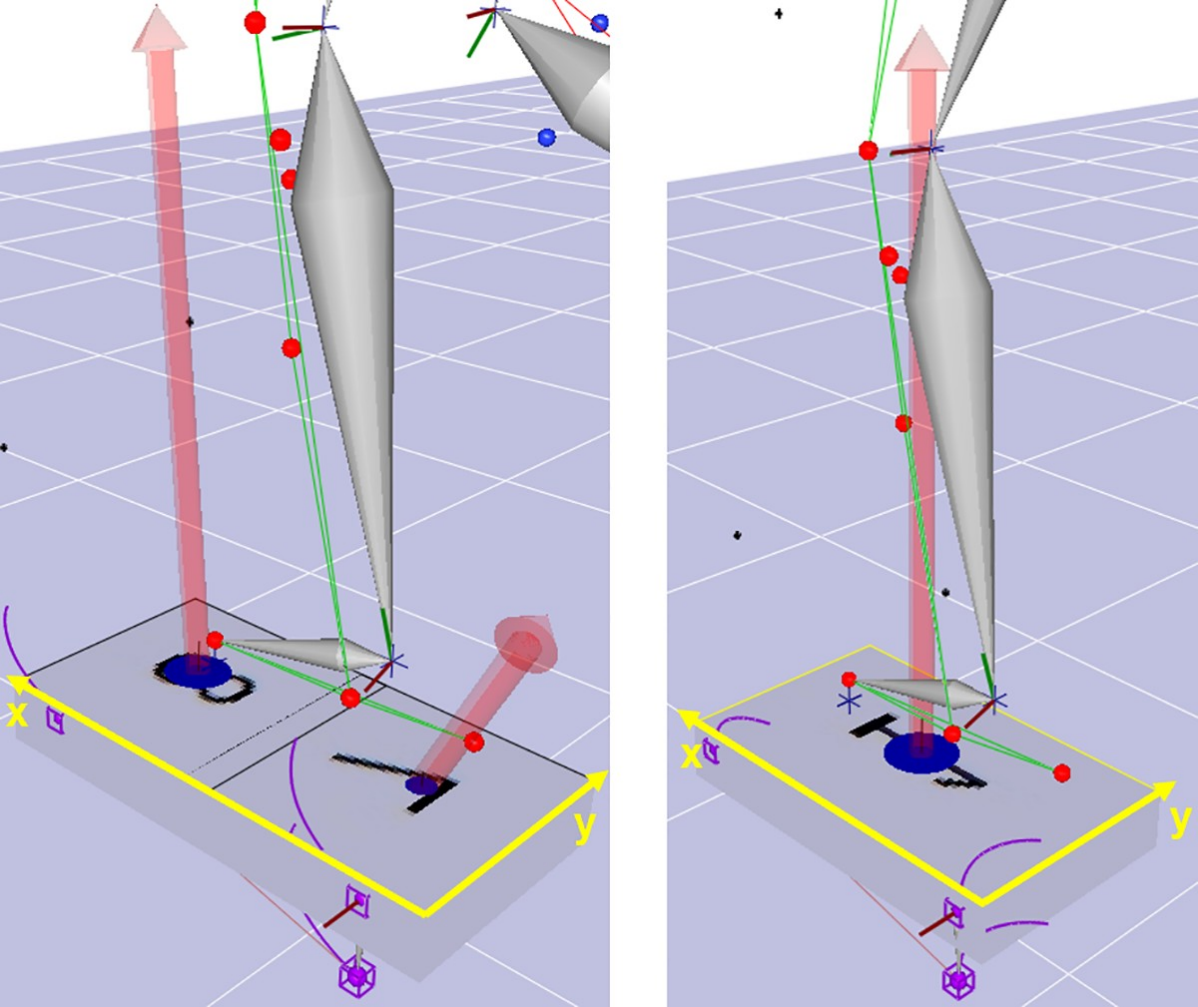
Elliptical force platforms in Cortex before (left) and after (right) combining load cell data into a resultant force-couple system.

Static and dynamic captures were processed in Cortex to define body segments used to calculate joint kinematics and kinetics. For cycling and elliptical trials, additional markers defined pedal segments which were necessary to track load cells relative to a global coordinate system and transform measured forces and torques to the foot coordinate system (Fig 2). For the elliptical, two load cells were attached to each pedal and their data were combined into an equivalent force-couple system using standard equations [44,45] in order to properly apply reaction forces to the foot (Fig 3).

Kinematic and kinetic data were captured in Cortex at 150 Hz. Marker trajectories were filtered in Cortex using a two-pass, 4^th^ order, zero phase shift Butterworth filter with a cutoff frequency of 6 Hz and reaction forces were filtered using the same filter with a 10 Hz cutoff frequency. Cutoff frequency for reaction forces was calculated using an optimal method [46]. Missing marker data were interpolating using a cubic spline in Cortex.

**Fig 4:**
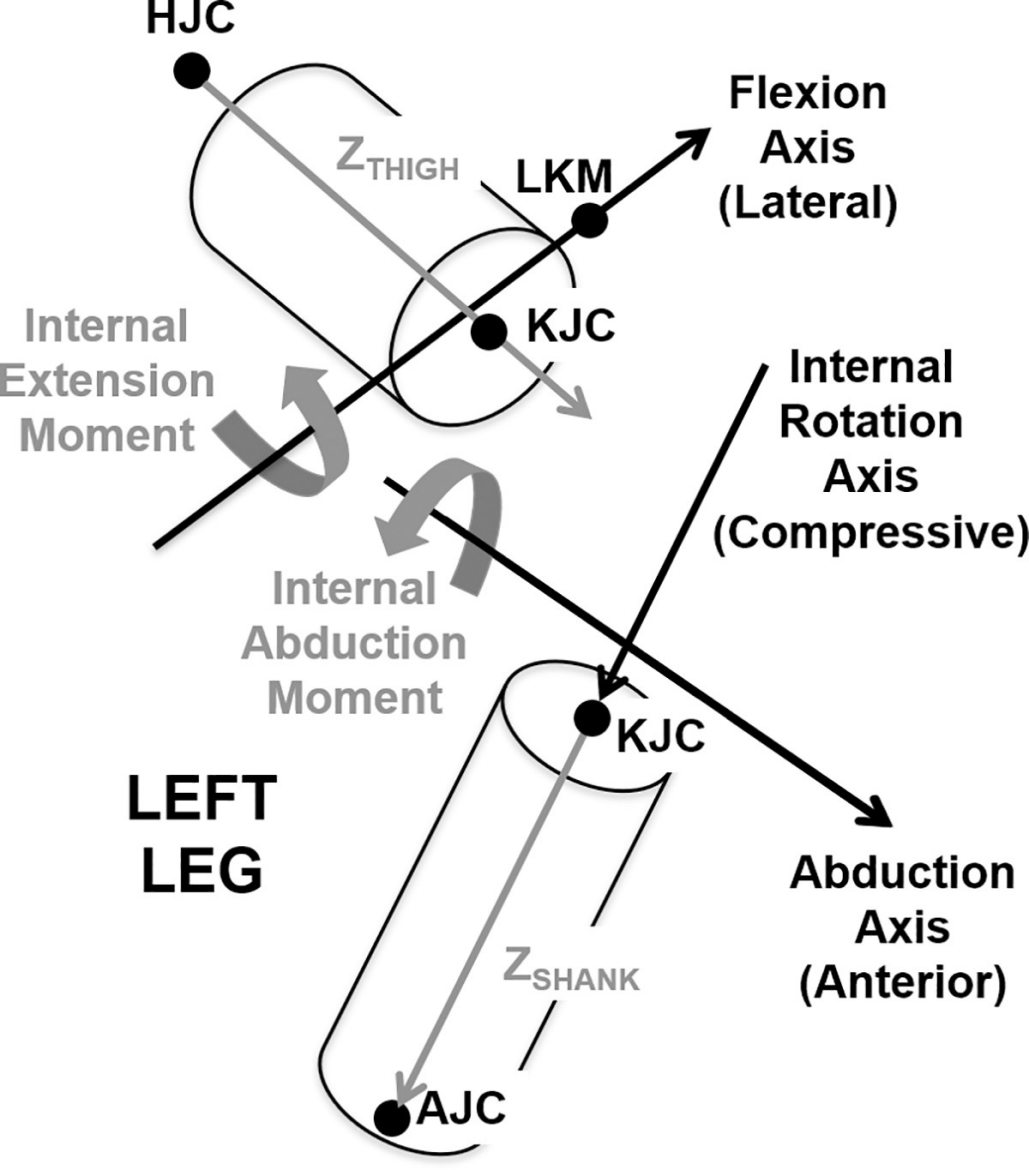
Floating axis knee joint coordinate system. Angle and force axes are indicated in the positive direction in black. Joint torques are defined in the positive orientation in gray. HJC is hip joint center, KJC is knee joint center, and AJC is ankle joint center. “Z” vectors indicate the longitudinal axis of the thigh and shank. The knee internal rotation axis is defined as the shank axis. The flexion axis is defined as the axis perpendicular to the thigh axis pointing through the lateral knee marker (LKM). The abduction axis is the cross product of the internal rotation axis and the flexion axis and points anteriorly.

Kinetics were calculated in Cortex using a bottom-up approach. The Cortex package KinTools RT used lower-body kinematics, standard shank and foot segment inertial properties [47], and measured reaction forces to calculate knee joint forces and torques via Euler’s equations [48]. The knee joint angles, resultant forces, and resultant torques for the knee were resolved into a floating axis joint coordinate system [49] (Fig 4). Knee flexion angle; lateral, anterior, and compressive force; and extension and abduction torque were defined to be positive. Reported torques follow the internal convention; that is, internal joint torques caused by muscle and joint contact forces oppose external torques due to ground or pedal reaction forces [50].

Analyses of kinematic and kinetic data results were performed using a custom script in MATLAB (MathWorks, Natick, MA, USA). All dynamic trials were interpolated to 101 data points from 0% to 100% cycle. Three trials for each participant in each exercise for each of the intact/dominant and residual/non-dominant legs were averaged. Knee kinematics and kinetics were then averaged across ten participants in each participant group.

Maximum knee flexion angles in gait, cycling, and elliptical for the intact/dominant and residual/non-dominant legs were normalized for each participant group by subtracting calculated knee joint flexion angles of the static pose from the dynamic angles measured during each trial [51]. Also, midstance and swing knee flexion angle maximums and corresponding timing in percent cycle in gait for amputee and control groups were analyzed. Forces were normalized by body weight and torques were normalized by body weight times height [52].

### Statistics

Analyzed data were tested for normality using Shapiro-Wilks tests and for equal variance using F-tests. Maximum knee compression force, extension torque, abduction torque, and maximum swing knee flexion angle in gait were found to be non-normal distributions and were analyzed using Kruskal-Wallis tests and post-hoc Dunn tests at 95% confidence with Bonferroni adjustments for multiple comparisons to analyze the differences between participant-leg-exercise groupings. Two-factor repeated measures analysis of variance (ANOVA) with post-hoc Tukey tests at 95% confidence were conducted to analyze the effects of participant and leg type (amputee intact leg, amputee residual leg, control dominant leg, control non-dominant) and exercise (gait, cycling, elliptical) on maximum knee flexion angle and to analyze the effects of participant and leg type (amputee intact, amputee residual, control dominant, control non-dominant) on knee flexion angle peaks and peak times in gait. All statistical analyses were performed in R [53]. A Bonferroni correction of four was applied (on account of four dependent variables) such that p<0.0125 was considered statistically significant.

## Results

The average amputee and control participant walking speeds were 1.26 ± 0.17 m/s and 1.29 ± 0.08 m/s, respectively. Average cycling RPMs were 69.45 ± 0.61 and 69.75 ± 0.61 for amputees and controls, respectively. Average elliptical RPMs were 70.32 ± 0.59 and 70.15 ± 0.54 for amputees and controls, respectively. A two-sample t-test showed that the walking speeds were not different (p = 0.566) between participant groups (t-tests were only run on gait speeds since they were self-selected).

**Fig 5:**
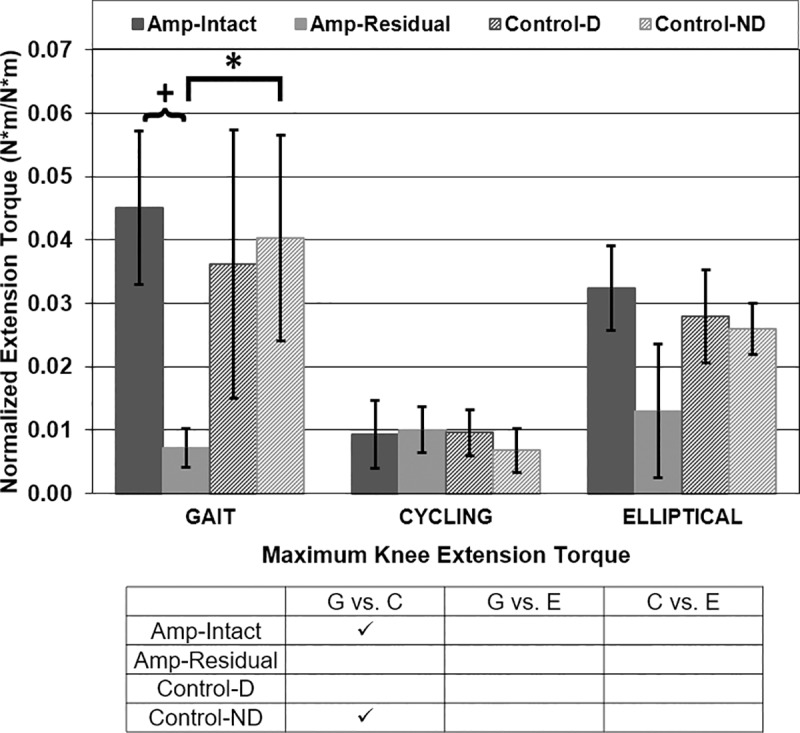
Maximum knee flexion angle [deg]. Mean ± 1 standard deviation. + = significance across leg type (intact/dominant vs. residual/non-dominant). * = significance across participant type (amputee vs. control). ✓ = significance across exercise type (gait vs. cycling vs. elliptical). P<0.0125 significant.

Maximum knee flexion angles were different between exercise types for all participant groups (Fig 5). P-values for maximum knee flexion angles between exercises (gait vs. cycling, gait vs. elliptical, cycling vs. elliptical) for all participant-leg combinations (amputee intact leg [Amp-Intact], amputee residual leg [Amp-Residual], control dominant leg [Con-D], control nondominant leg [Con-ND]) were all <0.001. Maximum knee flexion angles were asymmetrical for amputee participants in gait (p = 0.005) and elliptical (p<0.001) but not cycling (p = 0.014) (Fig 5).

**Fig 6:**
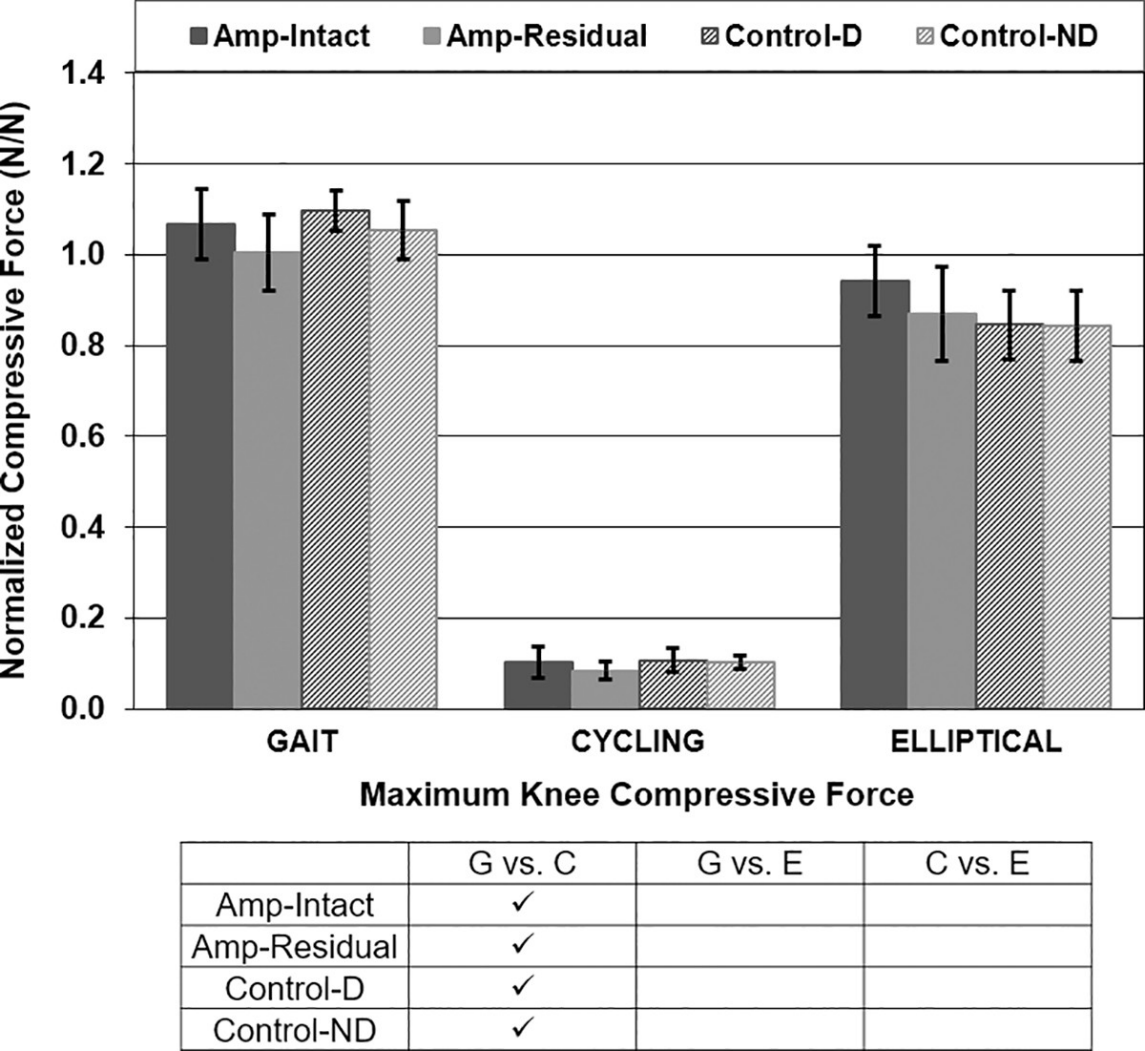
Maximum knee compressive force [N/N]. Mean ± 1 standard deviation. + = significance across leg type (intact/dominant vs. residual/non-dominant). * = significance across participant type (amputee vs. control). ✓ = significance across exercise type (gait vs. cycling vs. elliptical). P<0.0125 significant.

Maximum knee compressive forces varied in gait vs. cycling for all participant types and in cycling vs. elliptical for Amp-Intact legs (Fig 6). P-values for maximum knee compressive force comparisons in gait vs. cycling for all participant types were <0.001. Maximum knee compressive forces were not different between participant types or between intact/dominant and residual/non-dominant legs of either participant group (Fig 6).

**Fig 7:**
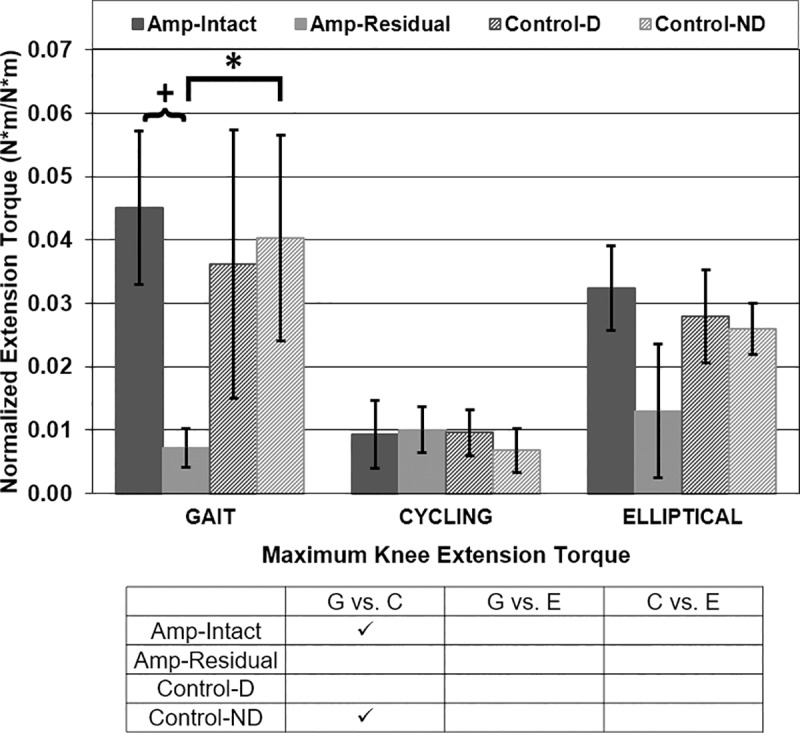
Maximum knee internal extension torque [Nm/Nm]. Mean ± 1 standard deviation. + = significance across leg type (intact/dominant vs. residual/non-dominant). * = significance across participant type (amputee vs. control). ✓ = significance across exercise type (gait vs. cycling vs. elliptical). P<0.0125 significant.

Maximum knee internal extension torque was different in gait vs. cycling for Amp-Intact and Con-ND legs and in cycling vs. elliptical for Amp-Intact legs (Fig 7). P-values for maximum knee extension torque comparisons in gait vs. cycling were <0.001 for Amp-Intact and Con-ND legs. Maximum knee extension torque for amputee participants was asymmetrical in gait (p<0.001) (Fig 7). Maximum residual/non-dominant knee extension torque was different between amputees and controls in gait (p<0.001) (Fig 7).

**Fig 8:**
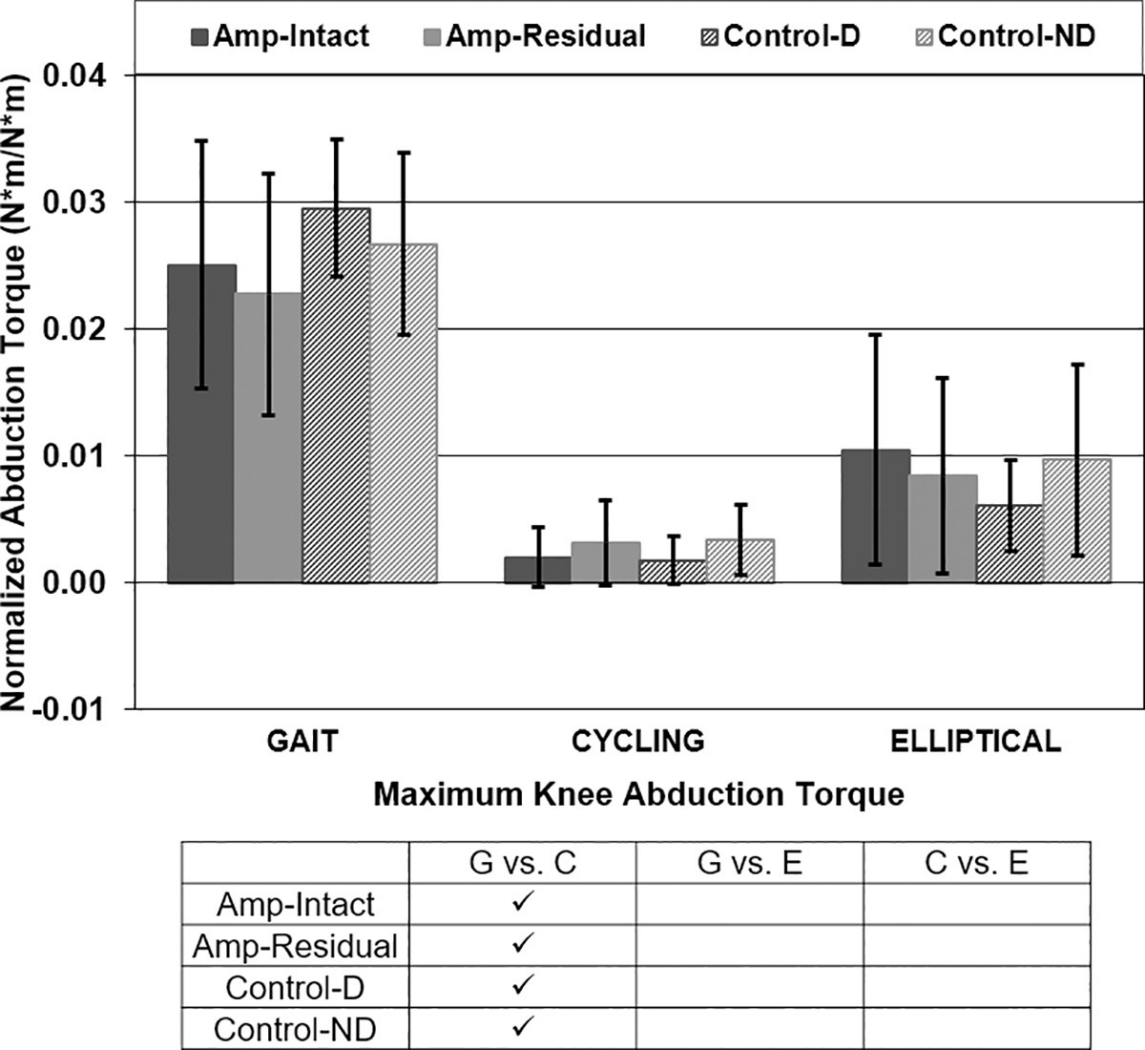
Maximum knee internal abduction torque [Nm/Nm]. Mean ± 1 standard deviation. + = significance across leg type (intact/dominant vs. residual/non-dominant). * = significance across participant type (amputee vs. control). ✓ = significance across exercise type (gait vs. cycling vs. elliptical). P<0.0125 significant.

Maximum knee internal abduction torque was different in gait vs. cycling for all participant types (Fig 8). P-values for maximum knee abduction torque comparisons in gait vs. cycling were <0.001 for all participant-leg combinations. Maximum knee abduction torque was not different between participant types or between intact/dominant and residual/non-dominant legs of either participant group (Fig 8).

**Fig 9:**
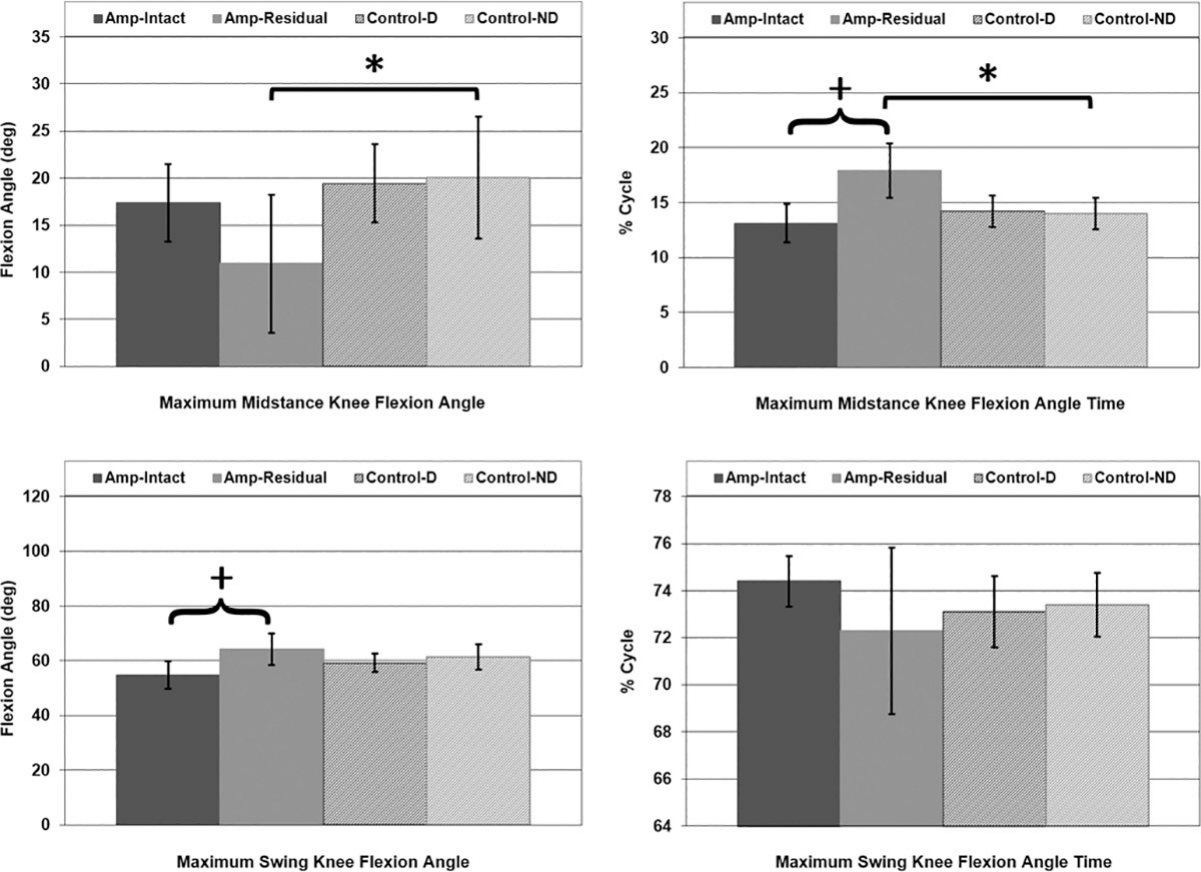
Maximum midstance and swing knee flexion angle [deg] and corresponding times [% Cycle] in gait. Mean ± 1 standard deviation. Midstance results are in the first row, swing results are in the second row, flexion angle results are in the first column, and flexion angle time results are in the second column. + = significance across leg. * = significance across participant type. P<0.0125 significant.

P-value for maximum midstance knee flexion angle in gait was 0.005 for Amp-Residual vs. Con-ND legs (Fig 9). P-values for midstance knee flexion angle timing were <0.001 for both Amp-Intact vs. Amp-Residual legs and Amp-Residual vs. Con-ND legs (Fig 9). P-value for maximum swing knee flexion angle in gait was 0.001 for Amp-Intact vs. Amp-Residual legs (Fig 9). No differences in swing knee flexion angle timing were found.

## Discussion

The hypotheses of the current study were: 1) knee biomechanics would differ due to participant status (amputee, control), exercise, and leg type (intact, residual) and 2) gait kinematic parameters would differ due to participant status and leg type. Kinematics and kinetics varied with exercise (all variables), leg type (flexion angle, extension torque), and participant type (extension torque in gait) providing support for the first hypothesis. Gait characteristics varied with leg type (midstance flexion angle timing, swing flexion angle) and participant type (midstance flexion angle, swing flexion angle) providing support for the second hypothesis. Knee joint compressive force, torques, and flexion angles were of similar magnitude to many other studies in both amputee and non-amputee populations in gait [16,17,21,54], cycling [55,56], and elliptical training [41,42,57] (see below for more details).

The kinematic results of the present study have clinical implications regarding rehabilitative and lifelong fitness sustainment exercises that may minimize OA risk in amputees. In the general population, abnormal knee kinematics are linked to the initiation of knee OA [2,58]. Specifically, knee flexion angle and timing are particularly linked to knee OA in the general population due to evidence that low midstance flexion angle and altered temporal characteristics will cause abnormal knee kinetics [59]. In this study, the amputee intact knee flexion angle was not different from that of control participants during the stance phase in gait. However, maximum residual knee flexion angle was significantly lower in midstance and had significantly delayed timing compared to the intact leg and the control non-dominant leg. Also, swing flexion angle was higher in the residual leg compared to the intact leg. These observed kinematic abnormalities in the residual leg may require compensation which could affect load bearing in the intact knee. For example, the observed reduction in residual knee midstance flexion angle could be evidence of an avoidance strategy (quadriceps-avoidance gait) also seen in anterior cruciate ligament (ACL) deficient populations that are at increased risk for intact knee OA [59]. Compensatory muscle activity in transtibial amputee gait, namely asymmetry in intact vs. residual knee flexor/extensor activity, has been found in previous studies [13,60].

Also, the kinetic results of the present study have similar clinical implications. Knee kinetics such as compressive force and muscle torques can be an indication of OA severity [60,61]; specifically, progression of cartilage degeneration is exacerbated by abnormal kinetics as increased cartilage compressive stress has been shown to be both a symptom of OA and a cause for further degeneration [2,58,61,62]. Abduction torque was found to have a particularly close link to the progression of knee OA and was of higher magnitude in cases of severe medial compartment OA [2,58,60,61,63] and tends to increase with increasing disease severity [26,64,65]. In the current study, no differences of amputee vs. control intact/dominant knee compressive force, extension torque, or abduction torque were found in any of the exercises. However, large asymmetries were present in maximum extension torque for amputees in gait suggesting that muscle coordination and braking/propulsion effort may be altered for the residual leg. Significantly reduced residual knee extension torque may be a sign that ESAR prostheses do not adequately replace natural biomechanics after amputation and may be causing the intact leg to compensate. Also, knee kinetics were generally lowest in cycling and highest in gait, suggesting that for populations at high risk for knee OA (such as amputees), cycling and other non-weight bearing exercises may be preferred for rehabilitation and lifelong fitness sustainment. However, this study did not directly link exercise type to injury or OA risk and a long-term study to identify evidence-based exercise guidelines for limiting OA risk is needed.

The results of the current study suggest that cycling may be an appropriate exercise for transtibial amputees whom are at high risk for knee OA. Specifically, the results suggest that exercises that constrain kinematics, such as cycling, are more likely to maintain typical cartilage loading patterns due to a lack of knee flexion angle and extension torque asymmetry as compared to gait and elliptical. The results also suggest that exercises that reduce overall knee joint forces and torques may be preferred for reducing OA risk. Cycling had generally lower magnitudes of resultant knee compressive force, extension torque, and abduction torque compared to elliptical training and gait. Other non-weight bearing exercises such as rowing or non-impact exercises such as stair-stepping may also be preferred over gait, while high-impact activities such as running and sports that involve foot planting (such as soccer) may increase the rate of OA progression due to high knee joint compression forces. Exercises for rehabilitation should, in general, minimize kinematic abnormalities and joint compressive stress to prevent or alleviate abnormal loading of cartilage but the present study did not relate exercise type to injury or OA risk. There is a clear need for a long-term study to associate injury risk with varying exercise protocols to produce specific exercise guidelines for limiting OA risk.

There are several limitations to the current study. One limitation of this work included marker-based errors which are common in motion analysis. Markers may have moved relative to the underlying bones due to soft tissue artifact (STA) and relative motion of compression clothing. STA, caused by marker movement due to skin deformation and displacement, affects the estimation of skeletal system kinematics with the exception of motion about joint flexion-extension axes [66,67]; this limitation was mitigated by only considering knee flexion angles. Markers defining the pelvis were placed on compression clothing; motion of these markers relative to the body would affect the calculation of the hip joint center which may impact knee flexion angle calculations; this limitation was mitigated by proper clothing fit. Another marker-based error, kinematic crosstalk, occurs when the calculated joint coordinate system is misaligned with anatomical axes which significantly influences knee joint abduction and internal rotation angles [68]; hence only knee flexion angles were considered. Another source of marker-based error was marker obstruction or loss during data collection; gaps in data were interpolated using a cubic spline.

A second limitation was inherent issues in any prosthetic assembly for amputee participants. While the same ESAR foot was used for all amputee participants the socket was not modified to enhance user experience and comfort with the prosthesis in exercises that amputee participants were not familiar with (cycling and elliptical). Socket-limb interfaces (such as liners, sleeves, or vacuum-based sockets) can permit motion of the prosthesis relative to the residual limb which can affect joint biomechanics. Future studies should make efforts to measure prosthesis motion relative to the residual limb.

In conclusion, transtibial amputees had significant asymmetry between intact and residual knee flexion angle in gait and elliptical and significantly reduced extension torque in the residual vs. intact knee in gait, whereas no asymmetries were detected for transtibial amputees in cycling. The results suggest that cycling, and likely other non-weight bearing exercises, may be a preferred exercise for limiting OA risk in transtibial amputees due to reduced asymmetry in knee kinematics and reduced knee kinetics as compared to gait values. Also, the results showed that state-of-the-art ESAR prosthetic design may not sufficiently restore amputee biomechanics to normal levels as evidenced by midstance knee flexion angle and peak extension torque asymmetry in gait and peak knee flexion angle asymmetry in elliptical. Muscle activation patterns could be responsible for altered residual knee flexion angle and extension torque in amputees and future work should involve EMG-driven inverse dynamics to calculate intact knee joint contact forces and muscle contributions. Since this study did not directly relate exercise type to injury or OA risk, there is a need for a long-term study that aims to directly associate injury or OA risk with varying exercise protocols for transtibial amputees and other populations at high risk for knee OA.
